# Enhanced Anticancer Activity of Nanoformulation of Dasatinib against Triple-Negative Breast Cancer

**DOI:** 10.3390/jpm11060559

**Published:** 2021-06-15

**Authors:** Fatemah Bahman, Valeria Pittalà, Mohamed Haider, Khaled Greish

**Affiliations:** 1 Department of Molecular Genetics, Kuwait Ministry of Health, Kuwait City 50000, Kuwait; fato88.fb@gmail.com; 2 Department of Drug and Health Science, University of Catania, 95125 Catania, Italy; 3 Department of Pharmaceutics and Pharmaceutical Technology, College of Pharmacy, University of Sharjah, Sharjah 27272, United Arab Emirates; mhaider@sharjah.ac.ae; 4 Department of Pharmaceutics and Industrial Pharmacy, Faculty of Pharmacy, Cairo University, Cairo 71526, Egypt; 5 Department of Molecular Medicine and Nanomedicine Unit, Princess Al-Jawhara Center for Molecular Medicine, College of Medicine and Medical Sciences, Arabian Gulf University, Manama 329, Bahrain

**Keywords:** TNBC, dasatinib, poly(styrene-co-maleic acid) micelles, nanoformulation, metabolism, EPR, nanomedicine, targeted therapy

## Abstract

Triple negative breast cancer (TNBC) is the most aggressive breast cancer accounting for around 15% of identified breast cancer cases. TNBC lacks human epidermal growth factor receptor 2 (HER2) amplification, is hormone independent estrogen (ER) and progesterone receptors (PR) negative, and is not reactive to current targeted therapies. Existing treatment relies on chemotherapeutic treatment, but in spite of an initial response to chemotherapy, the inception of resistance and relapse is unfortunately common. Dasatinib is an approved second-generation inhibitor of multiple tyrosine kinases, and literature data strongly support its use in the management of TNBC. However, dasatinib binds to plasma proteins and undergoes extensive metabolism through oxidation and conjugation. To protect dasatinib from fast pharmacokinetic degradation and to prolong its activity, it was encapsulated on poly(styrene-co-maleic acid) (SMA) micelles. The obtained SMA–dasatinib nanoparticles (NPs) were evaluated for their physicochemical properties, in vitro antiproliferative activity in different TNBC cell lines, and in vivo anticancer activity in a syngeneic model of breast cancer. Obtained results showed that SMA–dasatinib is more potent against 4T1 TNBC tumor growth in vivo compared to free drug. This enhanced effect was ascribed to the encapsulation of the drug protecting it from a rapid metabolism. Our finding highlights the often-overlooked value of nanoformulations in protecting its cargo from degradation. Overall, results may provide an alternative therapeutic strategy for TNBC management.

## 1. Introduction

Breast cancers are the top widespread type of tumor among females in the U.S., and in 2021, it is predicted that 280,000 new breast cancers will be diagnosed [1,2]. The disease is globally affecting about 1 in 8 women in the U.S. during their lifetime. Breast cancer mortality could be attributed to metastasis by 80–90% [3].

Triple negative breast cancer (TNBC) is a long-lasting orphan disease and among the most clinically challenging breast cancer subtype. TNBC is the most aggressive and heterogeneous breast tumor that lacks all of three therapeutically relevant biomarkers including estrogen receptor (ER), progesterone receptor (PR), and human epidermal growth factor receptor 2 (HER2) [4]. The conventional treatment for TNBC involves surgical excision and radiotherapy with a combination of adjuvant chemotherapies [5,6]. Despite current therapeutic regimens, patients affected by TNBC show frequently fatal prognosis and are exposed to early relapse and metastatic spread, as a result of resistance to chemotherapies [5]. Despite initially TNBC exhibiting more chemo-sensitivity than other groups of breast cancer, it shows high propensity to spread and metastasize to vital organs, for instance the lungs and brain, rendering the survival rate still significantly lower than patients with non TNBC across any phase of diagnosis [7,8,9]. These aggressive phenotypes can be at least to some extent ascribed to the incidence of breast cancer stem cells (BCSCs). In addition, the lack of targeted therapies increases the use of traditional chemotherapy often accompanied by severe side effects. The subclassification of TNBC based on gene expression profiling analysis includes basal like 1 (BL1) and basal like 2 (BL2), immunomodulatory (IM), mesenchymal (M), mesenchymal stem like (MSL), and luminal androgen receptor positive (LAR) [10,11,12]. This classification is paving the way to the identification of more specific molecular targets for TNBC treatment. In fact, these subtypes show different drug sensitivity profiles to anticancer treatments such as cisplatin for BL1 and BL2, PI3K, and proto-oncogene tyrosine-protein kinase (Src) inhibitors [13].

Src is a protein tyrosine kinase that regulates various cancerous events at an intracellular level, such as cellular adhesion, invasion, growth, survival, and vascular endothelial growth factor (VEGF) expression [14,15]. In addition, Src regulates an osteoclast function in normal bone and bone metastases [16]. A number of literature reports have evidenced in TNBC an abnormal activation and amplification of Src or Src-family kinases (SFK) and an involvement in metastasis regulation [17]. Not surprisingly, TNBC shows increased sensitivity to Src inhibitors compared to other cancer subgroups [18,19,20]. In addition, it has been demonstrated that ER and HER2 expression levels affect the beneficial effects of Src inhibitors in TNBC [21]. Therefore, Src can be considered as a new molecular target for TNBC therapy, and Src inhibitors have long been proposed as new antitumoral treatments, since they are able to prevent cell growth in liver, colon, breast, and ovarian cancers.

**Figure 1:**
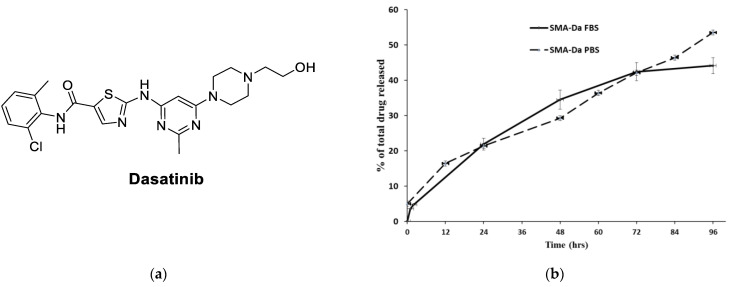
(**a**) Chemical structure of dasatinib; (**b**) SMA–dasatinib drug release studies. Cumulative release of the free drug from SMA–dasatinib micelles at pH 7.4 in PBS and FBS.

Dasatinib (Figure 1) is a Src, BCR-ABL, c-KIT, PDGFR-α and PDGFR-β, and ephrin receptor kinase blockers accepted by the Food and Drug Administration (FDA) for treating cases of Philadelphia chromosome positive leukemias (chronic myeloid leukemia; CML) [22,23]. Preclinical studies demonstrated significant inhibition of malignant breast cells growth through reducing the percentage of aldehyde dehydrogenase-positive (ALDH+) BCSCs within the BL-2 subtype of breast cancer [13,24]. Considering that BCSCs are often responsible for the onset of chemotherapy resistance, dasatinib has been considered for the treatment of TNBC [24,25]. Similarly, preclinical studies evidenced synergistic or additive dasatinib activity with chemotherapy, implying that this Src inhibitor can offer clinical benefit in TNBC [26]. Regrettably, patients suffering from TNBC have inadequate benefit from Src inhibitors treatment [27,28,29]. In fact, despite promising preclinical results, a phase II clinical trial by administering dasatinib as a single agent highlighted only a 9% clinical benefit rate, and other clinical trials terminated due to futility (e.g., NCT00817531, NCT00780676, etc.) [29]. Moreover, dasatinib suffers from some limitations related to its pharmacokinetic profile. Oral absorption of dasatinib is quick and produces around 80% of bioavailability; however, it is rapidly eliminated through CYP3A4-mediated metabolism, with a T_1/2_ of 3–4 h. In addition, dasatinib bioavailability has reduced the its ability to modify gastric pH value (antacids, H_2_-receptor blockers, proton pump inhibitors) and is modified according to the concomitant treatment with CYP3A4 inducers or inhibitors [30].

In recent years, considerable attention has been devoted to strategic application of nanoscience to pharmaceutical development with the aim of improving efficacy, delivery at the site of action, safety, physicochemical properties, and the absorption, distribution, metabolism, and excretion (ADME) profile of bioactive compounds [31]. In particular, nanoparticle formulations (NPs) can guarantee increased bioavailability of drugs administered orally, enhanced half-life of intravenous drugs (by reducing both metabolism and elimination), and augmented drug concentration in specific tissues [32]. Taking in account the dasatinib ADME profile, encapsulation of the drug into NPs may improve the drug efficacy, minimize side effects, and permit the active principle to assemble at the malignant tumor site by means of the enhanced permeability and retention (EPR) effect [33,34]. In addition, using the SMA micellar system to generate dasatinib NPs has multiple advantages over other nanoformulations. It produces a micelle with a nearly neutral or slightly negative charge reducing opsonization of the micelles, recognition by the reticuloendothelial system, and elimination from the blood circulation [35]. In our previous work utilizing dasatinib micelles compared to free drug, we had shown enhanced anticancer activity both in vitro and in vivo against various glioblastoma cell lines and in animal model of the disease [36]. In this study, we encapsulated dasatinib into polystyrene co-melic acid (SMA) micelles to generate micellar dasatinib system (SMA–dasatinib) that has been characterized for physicochemical properties including size, loading, charge, and release rate. In addition, SMA–dasatinib has been assessed for their anticancer effect in vitro using 4T1, MDA-MB-231, and MCF-7 cell lines and in vivo in a syngenic model of TNBC. The cell lines chosen represents a spectrum of commonly used breast cancer cell lines of both hormonal responsive and TNBC of human and murine origin. Our choice of cell lines will allow the comparison of dasatinib formulation in different biological environments and further allows the comparison of our results to earlier and subsequent research in the field. Encouraging obtained results will pave the way for further study in the management of TNBC.

## 2. Materials and Methods

Dasatinib were retained from LC Laboratories (Woburn, MA, USA). Polystyrene co-maleic anhydride (molecular weight~1600), Roswell Park Memorial Institute (RPMI) 1640 medium, Hank’s balanced salt solution, fetal bovine serum (FBS), bovine serum albumin (BSA), and TrypLE express were bought from ThermoFisher Scientific (Dubai, United Arab Emirates). *N*-(3-dimethylaminopropyl)-*N*-ethylcarbodiimide hydrochloride (EDAC), L-glutamine, antibiotic solution of penicillin/streptomycin were acquired from (Merck Hertfordshire, UK). All consumable materials including petri dishes, conical tubes (15 mL and 50 mL), cell culture flasks (25 and 75 cm^2^), and dialysis tubing were purchased from (Merck Hertfordshire, UK).

### 2.1. SMA–Dasatinib Micelles Synthesis

SMA–micelles were synthesized as previously reported [36]. Briefly, SMA was hydrolyzed by adding the SMA powder to 1 M NaOH solution at 70 °C to reach a concentration of 10 mg/mL. After this time, the pH of the obtained solution of SMA was adjusted to pH 5.0. This was followed by adding EDAC (1:1 weight ratio with SMA). Dasatinib was dissolved in dimethyl sulfoxide (DMSO) at 25% weigh ratio to SMA. Dasatinib was added to the solution, and pH was kept at 5 by adding 0.1 HCL until pH remained stable at 5.0. Then, the pH was raised up to reach 11.0 and kept until it become stable. The pH was then dropped to 7.4, and the solution was filtered 4 times by meand of a Millipore Labscale TFF system with a Pellicon XL 10 KDa cutoff membrane. Finally, the concentrated SMA–dasatinib micelles were frozen at −80 °C and the following day lyophilized (5 mTorr and −52 °C) to achieve a stable SMA–dasatinib powder.

### 2.2. SMA–Dasatinib Micelles Characterization

The SMA micelles loading was determined by using three different samples of 1.0 mg/mL of SMA–dasatinib micelles dissolved in DMSO for measuring absorbance at 320 nm of dasatinib to a previously prepared standard curve of the drug intending to determine the ratio between the micelle and the loaded dasatinib.

For size distribution and zeta potential determination of SMA–dasatinib micelles, a Malvern ZEN3600 Zetasizer Nano series was used (Malvern Instruments Inc., Westborough, MA, USA) by using 1 mg/mL of the SMA–dasatinib nanomicelles dissolved in both double DW as a solvent for size measurement or for charge measurement. Then, to measure the release rate of free drug (dasatinib) from SMA micellar system, two separate experiments have been performed by measuring the release in PBS and in FBS. A 2 mg of the SMA–dasatinib were dissolved in 2 mL of PBS or FBS, respectively, and inserted into a 10 kDa cutoff dialysis membrane that was flooded in 20 mL of PBS or FBS for 72 h. At specified time points, the surrounding water was collected from outside the dialysis bag and replaced with PBS or FBS, and the absorbance was measured at 320 nm.

### 2.3. Cell Culture

The cell lines 4T1, MDA-MB-231, and MCF-7 were obtained from American Type Culture Collection (ATCC) (Manassas, VA, USA). RPMI medium supplemented with 5% fetal bovine serum (FBS) was used to culture the cell lines while being maintained in a humidified atmosphere at 37 °C, 5% CO_2_.

#### In Vitro Anti-Proliferative Effect of Dasatinib and SMA–Dasatinib Micelles

Cells were seeded in 96-well plates (density: 4T1 5 × 10^3^, MDA-MB-231 5 × 10^3^, MCF-7 5 × 10^3^ cells/well, respectively) and incubated for 24 h at 37 °C in 5% CO_2_ and then treated with a different of concentrations of dasatinib 0 to 10 µM) or SMA–dasatinib (0 to 10 µM). The cytotoxicity was assessed after 48 h incubation using a sulforhodamine B (SRB) assay, as described previously [37]. Free SMA and DMSO at concentrations equivalent to that used for testing dasatinib were used as controls. Cells were fixed using 10% trichloroacetic acid and stained with SRB. The cytotoxicity experiments were performed in triplicate (*n =* 3). Then, the 50% growth inhibition (IC_50_) was assessed by using SRB assay after 48 h incubation. HepG-2 cells also were seeded at a density of 50,000 cells/cm^2^ onto a 25 cm^2^ flask. Then, cells were treated by various concentration of dasatinib and SMA–dasatinib. Twenty-four hours after incubation, the supernatants were collected and diluted accordingly to retreat 4T1 cells. Data were represented as mean ± SD of three independent experiments of each cell lines.

### 2.4. Effect of Dasatinib and DMA-Dasatinib Treatment in In Vivo Syngeneic Model

The Laboratory Animal Care Facility of the Arabian Gulf University (AGU), Bahrain, supplied the Female Balb/c mice (6–12 weeks old, mean weight 20–25 gm). Animals were maintained under standard conditions such as controlled temperature (25 °C), a 12 h photoperiod, and had access to food and drinking water ad libitum. All animal experiments were performed based on the rules and regulations of AGU Animal Care Policy and approved by the Research and Ethics Committee, REC approval No: G- E003-PI-04/17.

To propagate the tumor, female Balb/c mice (*n =* 3) were treated subcutaneously with 1 million 4T1 mammary carcinoma cells in both sides (right and left side) of the mice back. The tumor then was collected and cut down into small pieces of average size 1–3 mm^3^ in sterile PBS to sustain tumor viability. Following this, 5 mice of each group were cleanshaven, anesthetized, and injected with one small piece of the 4T1 tumor tissue subcutaneously. When the tumors reached 100 mm^3^ in size, mice were casually divided into three groups *n =* 5 in each group (negative control, dasatinib, and SMA–dasatinib) subjected to drug treatment. Dasatinib was given once at a dose of 5 mg/kg via the tail vein, whereas SMA–dasatinib at a dose of 5 mg/kg (dasatinib equivalent dose) dissolved in PBS was given by IV injection. The first day of drug treatment was established as day 0. Tumor volume was measured by manual caliber; the size was assessed by using this formula:

V (mm^3^) = ((transverse section (W)^2^ × longitudinal cross section (L))/ 2).

Tumor sizes were normalized by using the original tumor measure and represented as mean ± standard error of the mean (SEM). Additionally, the body weight of mice was estimated every day and normalized daily for 10 days.

### 2.5. In Vivo Biodistribution of Dasatinib and SMA–Dasatinib

Cells of 4T1 were injected into female Balb/c mice, bilaterally on the flanks to obtain 1–3 mm^3^ tumor size. When cancer volume reached 100 mm^3^, mice were casually divided into 2 groups (4 mice per group). Mice were injected with both dasatinib or the equivalent in SMA–dasatinib at 50 mg/kg via the tail vein. Mice were sacrificed 24 h after drugs injection and different organs were collected. Organs such as heart, lungs, liver, spleen, kidneys, and tumor tissue were examined for dasatinib content. SMA–dasatinib was taken out using the method reported earlier [38]. In brief, organs were crushed, weighed, and snap-frozen before pulverization. Obtained frozen tissue powder (1 mg) was treated with 67% ethanol and 1 mL of HCl 4M. The suspension was incubated at 70 °C for 30 min, sonicated, and centrifuged to take out dasatinib from tissue samples. Dasatinib amount was measured by absorbance at 332 nm and compared to a dasatinib calibration curve. The amount of dasatinib was standardized to the weight of tissue and to the whole weight of the organs from which it was extracted.

### 2.6. Statistical Analysis

The data from both experiments in vitro and in vivo were evaluated using GraphPad prism software. Tumor size measurements are expressed as group means ± SEM in the treatment groups. Cytotoxicity experiments with dasatinib and SMA–dasatinib are reported as means ± SD. The statistical significance of difference between groups were performed using a two-tailed t-test. Statistical differences were considered significant if the *p*-value was <0.05.

## 3. Results

### 3.1. Synthesis and Characterization of SMA–Dasatinib

SMA–dasatinib was synthesized and characterized by a low critical micelle concentration (CMC), as previously described [36]. Furthermore, the structural variation of hydrophobic styrene and hydrophilic maleic groups stimulates the quick construction of SMA micelles and facilitates the encapsulation of dasatinib. The loading of SMA–dasatinib was 11.5%, calculated as the weight ratio of the dasatinib over the total amount of SMA micelle weight. Micelles average size measuring showed that SMA–dasatinib micelles were 198 nm and had a polydispersity index (PDI) of 0.17, which was determined by dynamic light scattering (DLS). As shown in Table 1, the zeta potential of SMA–dasatinib is near neutral with a value of 0.0035 mV, which can sustain the micelle in the blood circulation for a long time by lowering the clearance by the reticuloendothelial system and allows accumulation in the tumor [39].

Thus, the average size of SMA–dasatinib is within the size range to facilitate its accumulation in tumor tissue by the effect of enhanced permeability and retention (EPR) [40]. Moreover, the release rate of the drug from the micelles was more efficient in an environment mimicking extracellular pH than in the blood (53 vs. 44%) following 96 h incubation).

The release of dasatinib from SMA micelles was assessed at physiological pH 7.4 in PBS and FBS, respectively, for 96 h (Figure 1). The SMA–dasatinib micelles were stable in solution with about half of the formulation released after 96 h. Moreover, in the first 2 h, the cumulative release was around 5%, as shown in Figure 1. The stability of the micellar system depends on the slow release in the blood circulation, which promotes the SMA–dasatinib accumulation at the tumor site through the EPR effect. A previous study has demonstrated the endocytosis of SMA micelle through caveolin-1 [41]. Therefore, SMA–dasatinib will be internalized by endocytosis and the release of dasatinib into the TNBC tumor cells.

### 3.2. Cytotoxicity of Dasatinib and SMA–Dasatinib versus Breast Cancer Cell Lines

The assessment of the cytotoxic effect of SMA–dasatinib and dasatinib on cell viability was achieved using different breast cancer cell lines, such as human MDA-MB-231, 4T1, and MCF-7 cells. A cell’s cytotoxicity of dasatinib and SMA–dasatinib was determined by means of the SRB assay. Equivalent concentrations of free SMA and DMSO were used to dissolve the dasatinib and yielded no cytotoxic effect.

**Figure 2:**
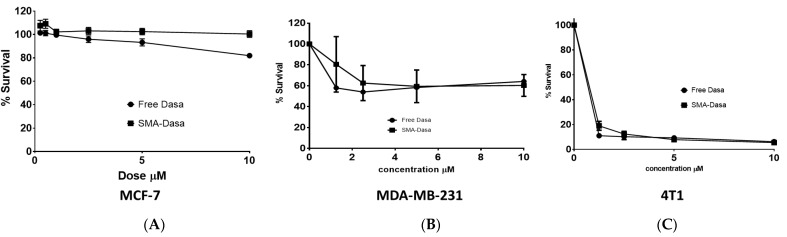
Cytotoxicity of dasatinib and SMA–dasatinib (**A**) against MCF-7, (**B**) MDA-MB-231, (**C**) and 4T1 cells. The cells were treated for 72 h with specific concentrations of dasatinib and SMA–dasatinib micelles. The cell number was determined using the SRB assay. Data are expressed as mean ± SEM (*n =* 3).

The treatment of MCF-7 cells (Figure 2A and Table 2) evidenced that either dasatinib and SMA–dasatinib showed no noteworthy difference in their cytotoxic activity after 48 h incubation and both displayed an IC_50_ > 10 µM. An IC_50_ value of 6.1 ± 2.2 µM was obtained for the dasatinib treatment of MDA-MB-231 cells, while SMA–dasatinib exhibited an IC_50_ value of 8.16 ± 3.1 µM (Figure 2B and Table 2). An enhanced effect could be partially attributed to greater internalization capability of MDA-MB-231 cells compared to MCF-7 cells [26].

Both MDA-MB-231 and MCF-7 reached a plateau, which can be explained by the inherent dependence of the breast cancer cell lines on tyrosine kinases signaling for growth and division. Cells of 4T1 treated with free dasatinib and SMA–dasatinib showed a significant cytotoxic effect when compared to MCF-7 and MDA-MB-231 cells with IC_50_ of 0.014 ± 0.003 and 0.083 ± 0.01 µM, respectively (Figure 2C and Table 2).

### 3.3. Effect of Dasatinib and SMA–Dasatinib on the Development of 4T1 Tumors

**Figure 3:**
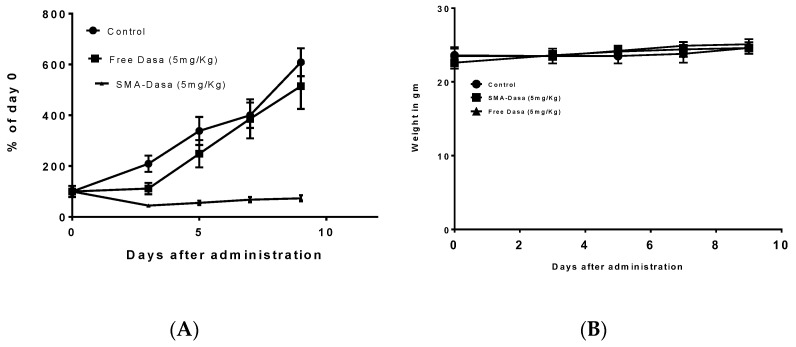
In vivo antitumor activity of dasatinib and SMA–dasatinib on 4T1tumor bearing Balb/c mice. Mice were treated for 10 days with single dose of either dasatinib 5 mg/kg and SMA–dasatinib 5 mg/kg. Control group was injected with PBS (pH 7.4). Tumor volume changes (**A**) and body weight changes (**B**) were monitored over the treatment period. Data are presented as the mean of triplicate experiments ± standard error.

The anticancer activity of dasatinib and SMA–dasatinib was evaluated using Balb/c mice harboring 4T1 tumor over a treatment period of 10 days. Figure 3A shows that during the first days’ treatment with free dasatinib (5 mg/kg) tumor growth seems to be delayed, while overall tumor size did not change significantly after 10 days in comparison to control-treated mice. Very differently, treatment with SMA–dasatinib almost entirely stopped the tumor growth for the duration of the study.

The therapeutic efficacy of dasatinib and SMA–dasatinib treatments were not associated with any statistically significant weight loss during the treatment period, as shown in Figure 3B and Table 3.

### 3.4. In Vivo Biodistribution of Dasatinib and SMA–Dasatinib

**Figure 4:**
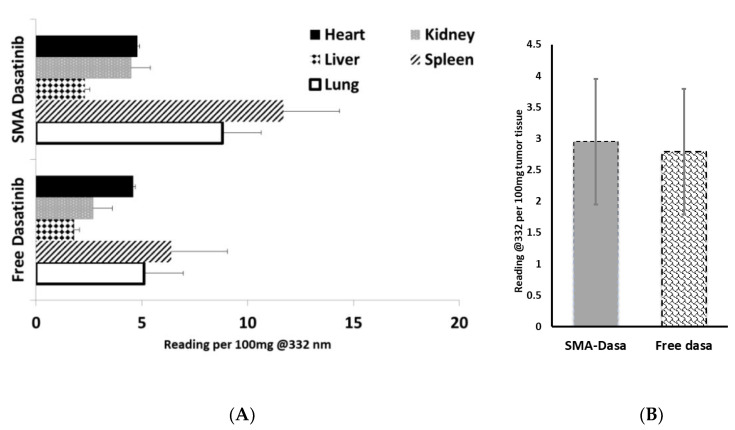
(**A**) Tissue and (**B**) tumor distribution of free dasatinib and SMA–dasatinib at 24 h after intravenous injection of dasatinib or SMA–dasatinib (50 mg/kg) to Balb/c mice bearing 4T1 tumors (*n =* 5). Representation of the relative content of dasatinib per 100 mg tissue expressed in free and micellar dasatinib.

The biodistribution of dasatinib and SMA–dasatinib were measured in vivo; to this extent, immunocompetent Balb/c mice harboring 4 T1 tumors were intravenously injected with equivalent doses of dasatinib or SMA–dasatinib, and the concentration of dasatinib in various organs and tumor has been measured. As reported in Figure 4, dasatinib and SMA–dasatinib are distributed to the heart, liver, lung, kidney, and spleen. There was an increased accumulation of dasatinib following SMA–dasatinib injection in the spleen, kidney, and lung when compared to the free dasatinib injection (Figure 4A). No significant statistical difference was observed in the heart and liver. Additionally, in the tumor when comparing SMA–dasatinib to the free dasatinib injection (Figure 4B), no statistically significant difference was observed.

### 3.5. Cytotoxicity of Dasatinib and SMA–Dasatinib Versus HepG2 Cell Line and 4T1 after Passage in HepG2

**Figure 5:**
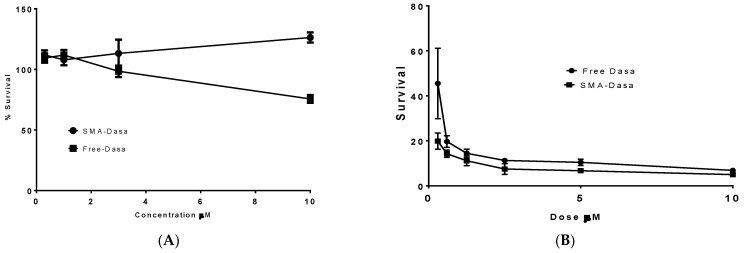
Cytotoxicity of dasatinib and SMA–dasatinib (**A**) against HepG2 cells, (**B**) 4T1 cells after treatment with HepG2. The cells were treated for 48 h with specific concentrations of dasatinib and SMA–dasatinib micelles. The cell number was determined using the SRB assay. Data are expressed as mean ± SEM (*n =* 8).

The effect of SMA–dasatinib and dasatinib on HepG2 cell viability was assessed by using SRB assay. HepG2 cells upon treatment with dasatinib and SMA–dasatinib micelles did not show any significant toxicity (Figure 5A) after 48 h incubation and both displayed an IC_50_ > 10 µM (Table 2). However, when the supernatants obtained from HepG2 treatment with dasatinib and SMA–dasatinib, respectively, were added to 4T1 cells the correspondent IC_50s_ varied noticeably. While the SMA–dasatinib cytotoxicity did not change (IC_50_ = 0.09 vs. 0.083 µM, respectively, Table 2); dasatinib cytotoxicity resulted in a 15-fold decrease in cytotoxicity (IC_50_ = 0.21 vs. 0.014 µM, respectively, Table 2).

## 4. Discussion

Dasatinib is a multi-target kinase inhibitor, including BCR/ABL kinases and Src family kinases (SFK) that are closely linked to multiple signal pathways that regulate proliferation, invasion, survival, metastasis, and angiogenesis [42]. Dasatinib showed promising results in the treatment of TNBC as a single agent or as a neoadjuvant; nevertheless, its use is limited by its poor aqueous solubility (6.49 × 10^−4^ mg/mL). Moreover, after oral administration, dasatinib is subjected to extensive first pass metabolism, where multiple CYP enzymes appear to have the potential to metabolize the drug [43]. Our current work aims at encapsulating dasatinib into SMA micelles to generate an SMA–dasatinib micellar system that can improve its solubility in water, protect the drug against enzymatic degradation, potentiate its chemotherapeutic effect, and minimize the rate of drug resistance.

The characterization of SMA–dasatinib micelles showed successful encapsulation of the drug with a loading capacity of 11.5%. Given that effective molecular size for EPR is 20–200 nm, the micellar size of 198 nm favors the accumulation of the nanoparticles in the tumor cells. In addition, the particle size of the prepared drug-loaded micelles should improve their circulation time and extend their plasma half-life by avoiding their rapid elimination from the kidney. The surface charge of the obtained prepared SMA–dasatinib micelles was almost neutrall, which is desirable to limit their interaction with active plasma constituents such as complement system and coagulation factors. Further, a near neutral charge will ensure selective EPR-based extravasation through tumor vasculature with minimal interaction with normal endothelial cell membrane. The micellar formulations showed a sustained slow-release rate of the drug for 96 h in both PBS and FBS (Figure 2), which shows that they can function as a reservoir for delivering a consistent level of dasatinib once concentrated extracellularly at tumor tissues and, hence, prolong the exposure of tumor cells to effective doses of the drug.

The examination of dasatinib-induced inhibition of metabolic activity on three commonly studied TNBC cell lines showed different responses. The MCF7 cell line was the least sensitive to treatment with SMA–dasatinib and free drug (IC_50_ > 10 µM) compared to MDA-MB-231 (IC_50_ 8.16 and 6.1 µM, respectively). This correlates with previous studies, which suggested that MDA-MB-231 are more sensitive due to the presence of active ABL kinase and their greater drug internalization capacity [26,44]. The 4T1 cell line exhibited a significantly high sensitivity to dasatinib and SMA–dasatinib (IC_50_ = 0.014 and 0.083 µM, respectively) compared to MDA-MB-231 and MCF7, which may be due to their sensitivity to Src (Kin-2) receptor tyrosine kinase blockade [45]. Interestingly, there was no significant difference in the cytotoxic effect of the SMA–dasatinib and the free drug on the different types of TNBC cell lines in vitro. Nevertheless, Figure 4 showed that treatment with SMA–dasatinib significantly inhibited the tumor growth in vivo compared to animals treated with the free drug. Both treatments resulted in no significant weight loss in treated animals, indicating that it is relatively safe to use dasatinib and SMA–dasatinib micelles in this animal model.

The biodistribution after IV administration showed a significantly high accumulation of SMA–dasatinib in the spleen compared to the free drug. This could possibly be due to the fact the size of SMA–dasatinib micelles is larger than the fenestration of the liver vasculature, which can reduce the hepatic uptake of the micelles and may decrease the metabolism of the drug. On the other hand, there was no significant difference between the tumor distribution of dasatinib and SMA–dasatinib. Dasatinib is characterized by a large volume of distribution and human plasma protein binding. In vitro studies showed that plasma protein binding of dasatinib can reach 96%, creating a depot from which the drug slowly releases its free form. It may also increase the molecular size of the drug and enhance its accumulation at the tumor site by EPR effect similar to SMA–dasatinib [46].

Treatment of HepG2 cells with dasatinib and SMA–dasatinib micelles did not show significant toxicity (IC_50_ > 10 µM). This is probably due to low expression levels of Src kinase, which reduced the sensitivity of the cell line to the drug [42]. The passage of dasatinib and SMA–dasatinib through HepG2 before treatment of 4T1 cells was carried out to check the effect of metabolism on the cytotoxic ability of the treatments. Dasatinib is significantly metabolized by CYP3A4 in the liver generating an active metabolite with similar potency to the drug; however, it represents only 5% of dasatinib in plasma. The co-administration of potent CYP3A4 inducer results in a considerable reduction on Cmax and AUC of the drug [43]. Treatment of 4T1 cells with supernatants obtained from HepG2 treatment showed a significant decrease in cytotoxicity of the free dasatinib, while the cytotoxic effect of SMA–dasatinib remained unchanged. The encapsulation of dasatinib offered protection for the drug against enzymatic degradation. The size of the produced micelles enhanced its accumulation at the tumor site by EPR effect and reduced its liver uptake. Our work is an emphasis of the overlooked advantage of nano-delivery systems in terms of cargo protection against degradation. This potential advantage was first described by Maeda back in 1991 [47]. Neocarzinostatin (NCS) is a very potent anticancer pretentious agent; however, NCS half-life is almost 1.9 min in tested mice. Using the nanoformulation of SMANCS protected the drug from the proteolytic activities in the plasma as well as extending its half-life by one order of magnitude. Further, this early work proved the safety of clinical use of SMA as a polymeric carrier for various biological payloads. Overall, our work further emphasizes the metabolic advantages of SMA–dasatinib nanosystems with a potential application for treating TNBC.

## 5. Conclusions

In this work, we have successfully synthesized and characterized an SMA nanomicellar system encapsulating the TKI dasatinib. Both the free drug and its nanoformulation have shown comparable cytotoxic activity in vitro against an array of breast cancer cell lines. The TKI and its nanoformulation proved to be more effective against TNBC cell lines compared to a hormone-sensitive cell line. In an animal model of 4T1 TNBC, the nanoformulations was about seven-fold more effective in controlling 4T1 implanted tumors. This pronounced in vivo activity was attributed to the protection of an SMA micellar system of TKI from the enzymatic degradation. Overall, our work can renew the interest in dasatinib as an effective treatment modality against TNBC.

## Figures and Tables

**Table 1:** Characterization of SMA–dasatinib ^1^.

Micelle	Recovery	Loading (wt./wt.)	Size (nm)	PDI ^2^	Zeta Potential (mV)
SMA–dasatinib	65%	11.5%	198	0.17	−0.0035

^1^ Data are shown as mean values ± standard deviation (SD). Values are the mean of triplicate experiments; ^2^ PDI = polydispersity index.

**Table 2:** Experimental IC_50_ values (μM) of free dasatinib and SMA–dasatinib towards human MDA-MB-231, 4T1, and MCF-7 cells.

Cell Line	IC_50_ (µM) ^1,2^	
	Dasatinib	SMA–Dasatinib
MCF7	>10	>10
MDA-MB-231	6.1 ± 2.2	8.16 ± 3.1
4T1	0.014 ± 0.003	0.083 ± 0.01
Hep-G2	>10	>10
4T1 after Hep-G2	0.21 ± 0.04	0.09 ± 0.012

^1^ IC_50_ value determination was performed using GraphPad Prism. Data are reported as IC_3_ values in μM ± standard deviation (SD). Values are the mean of triplicate experiments.^2^ The IC_50_ value calculations were calculated according to GraphPad prism algorithm and were included to have a numerical reference value of comparison, although it is clear that a plateau is reached after certain concentrations in MCF-7 and MDA-MB 231 cell lines.

**Table 3:** Body weight changes upon treatment with dasatinib and SMA–dasatinib were monitored over the treatment period ^1^.

Day	Control	Dasatinib	SMA–Dasatinib
0	23.5	23.5	22.6
9	24.6	25.1	24.6
Mean weight	23.8	24.2	23.9
Std. deviation	0.4637	0.7537	0.7987

^1^ Data are presented as the mean of triplicate experiments ± standard error.

## Data Availability

The data presented in this study are available on request from the corresponding authors.
